# Barriers and Enablers to Using a Patient-Facing Electronic Questionnaire: A Qualitative Theoretical Domains Framework Analysis

**DOI:** 10.2196/19474

**Published:** 2020-10-08

**Authors:** Janet Yamada, Andrew Kouri, Sarah-Nicole Simard, Stephanie A Segovia, Samir Gupta

**Affiliations:** 1 Daphne Cockwell School of Nursing, Faculty of Community Services Ryerson University Toronto, ON Canada; 2 Department of Medicine University of Toronto Toronto, ON Canada; 3 Division of Respirology St. Michael's Hospital, Unity Health Toronto Toronto, ON Canada; 4 Keenan Research Centre for Biomedical Science St. Michael's Hospital, Unity Health Toronto Toronto, ON Canada

**Keywords:** asthma, electronic questionnaire, patients, barriers, enablers, mobile phone

## Abstract

**Background:**

Electronic patient questionnaires are becoming ubiquitous in health care. To address care gaps that contribute to poor asthma management, we developed the Electronic Asthma Management System, which includes a previsit electronic patient questionnaire linked to a computerized clinical decision support system.

**Objective:**

This study aims to identify the determinants (barriers and enablers) of patient uptake and completion of a previsit mobile health questionnaire.

**Methods:**

We conducted semistructured interviews with adult patients with asthma in Toronto, Canada. After demonstrating the questionnaire, participants completed the questionnaire using their smartphones and were then interviewed regarding perceived barriers and enablers to using and completing the questionnaire. Interview questions were based on the Theoretical Domains Framework to identify the determinants of health-related behavior. We generated themes that addressed the enablers and barriers to the uptake and completion of the questionnaire.

**Results:**

In total, 12 participants were interviewed for saturation. Key enablers were as follows: the questionnaire was easy to complete without additional knowledge or skills and was perceived as a priority and responsibility for patients, use could lead to more efficient and personalized care, completion on one’s own time would be convenient, and uptake and completion could be optimized through patient reminders. Concerns about data security, the usefulness of questionnaire data, the stress of completing it accurately and on time, competing priorities, and preferences to complete the questionnaire on other devices were the main barriers.

**Conclusions:**

The barriers and enablers identified by patients should be addressed by developing implementation strategies to enhance e-questionnaire use and completion by patients. As the use of e-questionnaires grows, our findings will contribute to implementation efforts across settings and diseases.

## Introduction

### Background

Asthma affects approximately 7.7% of adults in the United States and carries an annual economic burden exceeding US $50 billion [1,2]. As in many other common chronic diseases, large gaps between guideline-recommended care and real-world care continue to exist in asthma [3,4].

Patient-facing electronic health questionnaires represent a promising strategy to address clinical care gaps, as they engage patients in taking a more active role in their care, increase efficiency by reducing data acquisition burdens on clinicians, and collect data that can be directly processed through computerized decision support system (CDSS) algorithms and then integrated into point-of-care electronic health records (EHRs) for clinicians [5]. With the dramatic rise in smartphone ownership in the United States [6] and a shrinking *digital divide* [7], mobile health (mHealth) previsit questionnaires have emerged as a commonly used strategy across primary and specialty care settings [8-11]. However, little is known about patient perceptions of these questionnaires and their actual uptake and what strategies can be leveraged to drive their use. It is of great importance to understand the user’s perceived barriers and enablers to use mHealth technology to maximize its success [12].

Our team developed the Electronic Asthma Management System (eAMS), a point-of-care CDSS, to bridge key care gaps in the management of asthma in primary care settings [5]. The eAMS consists of a previsit electronic patient questionnaire that collects asthma-related parameters and a CDSS that receives and processes questionnaire data to produce guideline-based decision support (asthma control level, corresponding medication change recommendations, and a self-management tool called an asthma action plan (AAP) [4] integrated into the EHR in real time). In a 2-year interrupted time series study involving 890 patients and 237 clinicians, the eAMS significantly improved the frequency of asthma control assessment, the ratio of controller to reliever medication prescriptions, and delivery of AAPs to patients [5]. However, the previsit questionnaires were used by only 61.2% (551/890) of patients [5]. A detailed uptake analysis identified several quantitative predictors of questionnaire uptake [13].

### Objectives

In this study, we sought to identify the determinants (barriers and enablers) of uptake and completion of the eAMS mHealth previsit patient questionnaire, with a view to tailoring targeted strategies to overcome barriers and leverage enablers to drive patient usage. Given the growing popularity of mobile patient questionnaires, we identified themes that would be broadly relevant across such systems.

## Methods

### Recruitment

This was a qualitative study. Patients were recruited from the St. Michael’s Hospital’s Respirology Patient Database via telephone or email, and each provided informed written consent. We included adults (aged ≥18 years) with a clinical diagnosis of asthma, who were able to speak and write English, and who owned a smartphone. Each participant received a Can $50 (US $38) gift card for participating in the study. Ethics approval for the study was obtained from the Research Ethics Boards at Ryerson University and St. Michael’s Hospital.

### Sample Size

For theory-based interview studies, Francis et al [14] recommended a minimum of 10 interviews, followed by an initial analysis, and up to an additional 3 interviews or fewer if data saturation (ie, no new themes generated) is achieved before that. Accordingly, we sought to interview at least 10 participants and up to 3 more pending interim analyses for data saturation.

### Interview Procedure

All interviews were conducted by 3 members of the research team trained in conducting qualitative interviews. Interviews were audio recorded and transcribed verbatim. After consent was obtained, participants were asked to complete a data collection form for background information (demographics, asthma-related information, and smartphone usage). Given that group sessions featured direct use of the eAMS questionnaire, each participant was asked to bring his or her own smartphone and was emailed a link to the questionnaire at the start of the session. We then explained and demonstrated the functions, role, and workflow of the questionnaire (including information transmission to the clinician’s CDSS) and asked participants to independently complete the questionnaire on their device.

The questionnaire takes 5 to 10 min to complete and poses a series of questions that determine guideline-based asthma control levels, identify the individual’s asthma symptoms and triggers (eg, sports, activities, location), and elicit current medication use. We created a web-responsive version of the tablet-based waiting room questionnaire that was used in the original eAMS study [5]. The original questionnaire’s design and testing process are described in detail elsewhere [15,16]. Briefly, the questionnaire was designed based on the best principles for touch questionnaire usability [16], and both its content and usability were optimized through patient feedback in a rapid cycle design process (with summative qualitative analysis) involving 20 patients with asthma sampled purposively to represent a broad range of ages and electronic technology and touch device experience. The questionnaire achieved an *excellent* System Usability Scale score of 84.2 (SD 14.7). The System Usability Scale is a 10-item Likert scale questionnaire that has been used widely to measure the perceived effectiveness of, satisfaction with, and system efficacy of technological apps [15-17].

Immediately after completing the questionnaire, participants were asked specific questions related to the barriers and enablers to complete the questionnaire in a real-world setting. The described real-world workflow included downloading an app to their smartphone or tablet and accessing the questionnaire through this app for up to 1 week before their doctor’s appointment (up to and including in the waiting room immediately before the appointment). The interview guide was based on the Theoretical Domains Framework (TDF), an integrative framework comprised of 14 theoretical domains derived from 33 validated health and social psychology theories and 128 constructs that may drive and explain health-related behavior change from a psychological perspective [18,19]. The TDF is a comprehensive approach to understanding the determinants of behaviors in health care professionals and patients; it has been applied successfully for this purpose across diverse diseases and health care settings; and it has emerged as a standard for barrier and enabler measurement in implementation research [20]. The 14 theoretical domains include (1) knowledge; (2) skills; (3) social/professional role and identity; (4) beliefs about capabilities; (5) optimism; (6) beliefs about consequences; (7) reinforcement; (8) intentions; (9) goals; (10) memory, attention and decision processes; (11) environmental context and resources; (12) social influences; (13) emotions; and (14) behavioral regulation [20]. The interview guide was designed to explore which domains in the TDF were relevant for the targeted behavior (ie, completion of the eAMS questionnaire before the clinical visit) and how each of those domains influenced this behavior [20]. Table 1 describes the 14 TDF domains [19] and associated interview questions.

**Table 1:** Theoretical Domains Framework: theoretical domains and associated questions.

Theoretical Domains Framework domain	Definitions	Example interview questions
Knowledge	An awareness of the existence of something	Having seen the questionnaire, do you need more information? What would need to be in place for you to do this questionnaire before seeing your doctor?
Skills	An ability or proficiency acquired through practice	What types of training, if any, would have been required to help you to complete the questionnaire?
Social/professional role and identity	A coherent set of behaviors and displayed personal qualities of an individual in a social or work setting	Do you feel it is part of your responsibility to complete this questionnaire? Do you think this is this something you should do?
Beliefs about capabilities	Acceptance of the truth, reality, or validity about an ability, a talent, or a facility that a person can put to constructive use	How easy or difficult was it to complete the asthma questionnaire? What parts of the questionnaire were easy or difficult to complete? How confident were you in your ability to complete the questionnaire?
Optimism	The confidence that things will happen for the best or that desired goals will be obtained	How likely do you think it is that completing the asthma questionnaire would lead to better management of your asthma by your doctor?
Beliefs about consequences	Acceptance of the truth, reality, or validity about outcomes of a behavior in a given situation	What do you think are the benefits of completing the asthma questionnaire? What are the good things that can happen as a result of completing the asthma questionnaire?
Reinforcement	Increasing the probability of a response by arranging a dependent relationship, or contingency, between the response and a given stimulus	Are there any good experiences that you have had with your asthma management that would increase your likelihood of completing the asthma questionnaire?
Intentions	A conscious decision to perform a behavior or a resolve to act in a certain way	Is completing the questionnaire something you plan to do if you are asked to complete the questionnaire in the future?
Goals	Mental representations of outcomes or end states that an individual wants to achieve	How much of a priority would it be to complete the asthma questionnaire before you see your doctor, compared to other priorities?
Memory, attention and decision processes	The ability to retain information, focus selectively on aspects of the environment, and choose between 2 or more alternatives	Is completing a questionnaire before seeing a doctor something you usually do? Would you remember to complete the questionnaire? When could you see yourself forgetting to complete the questionnaire? Why? What could help to prevent this? Would a text or email message 1 week before your appointment be a sufficient reminder?
Environmental context and resources	Any circumstance of a person’s situation or environment that discourages or encourages the development of skills and abilities, independence, social competence, and adaptive behavior	Is there anything that might influence whether or not you would complete the questionnaire? (eg, Wi-Fi availability, equipment to complete, links broken, time, competing tasks, etc)
Social influences	Those interpersonal processes that can cause individuals to change their thoughts, feelings, or behaviors	Who would influence you to complete the questionnaire? (eg, family member, doctor, etc)
Emotions	A complex reaction pattern, involving experiential, behavioral, and physiological elements, by which the individual attempts to deal with a personally significant matter or event	Tell me how you feel about completing the asthma questionnaire before you see your doctor. Are you worried, or do you have any concerns about completing the questionnaire?
Behavioral regulation	Anything aimed at managing or changing objectively observed or measured actions	Can you think of a plan for how you would ensure that you complete the asthma questionnaire before your appointment?

### Data Analysis

Participant characteristics were summarized in detail. In total, 2 trained qualitative analysts (JY and SS) independently coded transcripts into the 14 TDF domains (using directed content analysis) [21]. They first coded 2 transcripts independently and together defined a consensus coding scheme (ie, codes, definitions of codes, examples of quotes under each code). This coding scheme was used to independently code the remaining transcripts (using NVivo 12 software, QSR International). The coders met on a regular basis to discuss discrepant codes, which were resolved through consensus. For each coded quote, the analysts generated a statement that reflected the core belief expressed by that response (a *belief statement*) [20]. These statements were grouped into themes that suggested significant influence on the uptake of the questionnaire. In instances where a single domain allocation agreement could not be reached, the statement was placed in both pertinent domains [20]. Emerging themes and belief statements were verified by each analyst and adjusted for consensus. To ensure the trustworthiness of the data, the analysts used memos during the coding process and developed an audit trail to track progress and enable easy reference to the primary data at a later time, if necessary. TDF domains that were considered of high importance were those with (1) belief statements in the domain that had relatively high frequencies (ie, more than one-third of respondents identified the belief), (2) conflicting beliefs in the domain (ie, where participants identified opposing beliefs), and (3) evidence of strong beliefs in a domain that is believed to directly impact uptake (as determined by the consensus of research team members) [20,22]. Verbatim quotes along with their categories and themes were used to support the belief statements.

## Results

### Overview

Data were collected from June 2018 to April 2019. Of the 21 patients who were approached to participate, 12 were recruited and consented to be interviewed. We performed an interim analysis after the first 10 interviews and coded each transcript thereafter until saturation. Saturation was achieved after another 2 interviews, for a total of 12 interviews.

### Participant Characteristics

Participant characteristics are summarized in Table 2. All participants were aged ≥31 years, 58% (7/12) of the participants were female, and a majority had a college education or higher. All participants used their smartphones frequently during the day for a variety of purposes. Each face-to-face interview lasted approximately 45 min.

**Table 2:** Participant characteristics (n=12).

Variable		Values
**Age (years), n (%)**		
	25-30	0 (0)
	31-40	3 (25)
	41-50	3 (25)
	51-60	2 (17)
	≥60	4 (33)
**Sex, n (%)**		
	Female	7 (58)
**Highest level of education completed, n (%)**		
	University	8 (66)
	College or trade school or other	2 (17)
	High school	2 (17)
	Elementary school	0 (0)
Number of years since asthma diagnosis, median (IQR)		8 (5.25-36)
Inhaler medication for asthma, n (%)		12 (100)
**Use of rescue inhaler, n (%)**		
	Yes	8 (66)
	No	2 (17)
	Not reported	2 (17)
**Use of controller inhaler, n (%)**		
	Yes	11 (92)
	No	1 (8)
**Type of smartphone, n (%)**		
	Blackberry OS^a^	1 (8)
	iPhone	6 (50)
	Android OS^b^	5 (42)
**Smartphone activity (check all that apply), n (%)**		
	Email	10 (83)
	Recreation (eg, text messaging)	10 (83)
	Information seeking (eg, finding addresses, directions)	9 (75)
	Information storage (eg, contacts)	9 (75)
	Scheduling (eg, to-do lists, appointments)	8 (66)
	Other (eg, Facebook, GPS)	6 (50)

^a^OS: operating system.

^b^Android OS: included phones manufactured by Samsung, Huawei, LG, and OnePlus.

### Relevant Theoretical Domains

Participants spent an average of 9.6 (SD 5.2) min to complete and review the questionnaire before the interview. All 14 TDF domains were identified as relevant. Most of the belief statements focused on enablers to use the questionnaire. A summary of the 14 TDF domains, related belief statements, and participant quotes is provided in Multimedia Appendix 1.

### Enablers

Overall, the majority of participants indicated that the eAMS questionnaire was easy and straightforward to complete, with no training required (knowledge, beliefs about capabilities, and skills). Most participants stated that they would complete the questionnaire on their phone if they were given that option (intention). Completion of the questionnaire was viewed by participants as important and a priority (goals). Having the option to complete the questionnaire on their own time was considered a benefit to participants (environmental context and resources):

I think it’s more relaxed which makes the patient feel easier and there’s no rush. Like okay, you’re doing the questionnaire and somebody comes to the door or you can just go answer the door, and then go back to the questionnaire, or be doing the questionnaire at 3 o’clock in the morning if you want…

P1003

Several participants reported that their prior experiences with poor disease control motivated them to use the tool (reinforcement), and the majority of participants reported that it was their responsibility to complete the questionnaire to enhance their level of care (social/professional role and identity):

I feel it is [my responsibility] because the way I look it, the more information my doctor has about what’s going on with me, the better he or she is able to help me manage my symptoms and cope.

P1004

Most participants described benefits to completing a patient questionnaire before their medical appointment, including that their primary care physician would have a more thorough understanding of their condition and that this would translate into better health care and disease control (eg, this would inform their self-management plans; beliefs about consequences). Participants also indicated that completing the questionnaire would enhance their understanding of their own disease control (beliefs about consequences):

I think from the patient’s perspective, it would give them more insight as to what’s happening because they actually have to think about what their symptoms are and what they’re doing to help the asthma, so therefore, they can bring that information to the doctor.

P1008

More than half of the participants felt that receiving a reminder from the doctor’s office via email, text, or phone before their appointment would ensure that the questionnaire would be completed (behavioral regulation, memory, attention and decision processes; environmental context and resources).

### Barriers

Several participants mentioned that competing priorities might influence whether they would complete the questionnaire in advance of their medical appointment (environmental context and resources):

I have my two personal emails. I have 4 different work emails that I have to go through every day. Like it’s swamped with these things.

P1003

Some would have preferred to complete the questionnaire on a larger device (eg, computer, iPad; environmental context and resources) or on paper. Barriers to using the questionnaire also included conflicting beliefs. A small number of participants felt that completing the questionnaire would not help them to understand their asthma nor lead to better asthma control (beliefs about consequences). Several participants expressed concerns about web-based security of questionnaire data and whether they could complete the questionnaire correctly (emotions).

## Discussion

### Principal Findings

To our knowledge, this is the first study to formally assess barriers and enablers to patient use of an mHealth questionnaire and the first to apply the validated TDF for this purpose. In identifying the determinants of uptake of the eAMS patient questionnaire, we identified constructs that are likely applicable to mHealth questionnaires across diseases.

First, we noted that factors relating to the content, format, and usability of the questionnaire itself were mostly perceived favorably and characterized as enablers to its use, as opposed to barriers. Specifically, most users did not believe that they needed extra training in the form of external knowledge or skills to complete the questionnaire (knowledge and skills) and clearly indicated that they were capable of completing it as required (beliefs about capabilities). This is likely a direct consequence of the user-centered approach taken in questionnaire development, whereby both content and format were serially improved in response to user feedback in focus group settings [15,16]. Indeed, Sun et al [23] previously reported that perceived ease of use is positively associated with behavioral intention to use mHealth systems. Accordingly, our findings reinforce the value of the integrated knowledge translation approach [24] in general and of rapid cycle design in particular, whereby end user engagement at an early stage of development is critical to ensure that the intrinsic features of a tool are conducive to later uptake [25]. Yet, it should be noted that 2 participants indicated that some users might require telephone or in-person support during questionnaire use (environmental context and resources), and it is likely that universal and sustainable adoption of any such mobile questionnaire will require investment in some form of technical support for a small number of users [26], even if they own a smartphone. As noted, the questionnaire completion rate was only approximately 60% in our previous study, which required patients to complete the questionnaire on a waiting room–based tablet [5]. In this study, the use case involved completion on their own device, up to 1 week in advance of the appointment, and patients indicated that both the ability to complete it on their own time schedule and in their own physical space were facilitators (environmental context and resources). This preference for e-questionnaire completion at home as opposed to within a health care facility was also noted in a previous study [27]. This suggests that questionnaire modalities that are limited to the waiting room environment, such as tablets or kiosks, may be less favorable. However, the advance completion approach would not be able to leverage typical prompts and reminders used in the waiting room environment (both human [eg, receptionist] and visual [eg, waiting room posters]). Correspondingly, some patients believed that they might fail to complete the questionnaire because of barriers presented by a lack of time or competing day-to-day priorities (environmental context and resources), and others indicated that they might simply forget (memory, attention and decision processes). Although some patients noted that they could set a calendar or personal email or device reminder (behavioral regulation), the vast majority indicated that a *friendly* (external) reminder would be a key facilitator (memory, attention and decision processes), with preferred modalities being email or SMS text message (environmental context and resources). In summary, the balance of our findings suggests that completion at home on a personal device is preferable but that electronic reminders should also be incorporated into any e-questionnaire implementation plan. Given that health practitioners were the most commonly reported influencers who could drive usage (social influences), invitations and reminders should ideally be addressed from the patient’s own health care provider.

As for the mode of delivery of the questionnaire, most users preferred the electronic system to a conventional paper-based questionnaire, although it is of note that 2 participants would have preferred paper (environmental context and resources). Several previous studies have similarly reported that most patients, across conditions, prefer an e-questionnaire to a paper-based one. [28-30]. Barentz et al [29] found that a preference for e-questionnaires was greatest in younger and more educated patients. Although our study included participants across a wide age spectrum, most were highly educated (10/12, 83% had a college education or higher), which may have contributed to this finding. It is also of note that some participants would prefer using a larger device, such as a personal computer or laptop (environmental context and resources). This supports previous observations that mobile apps should not exist in isolation [26]; any such mHealth questionnaire should be complemented by both a conventional website and tailored or web-responsive content accessible across platforms [26].

The vast majority of participants in our study felt that it was part of their responsibility to complete the questionnaire before their appointment (social/professional role and identity). Indeed, given a gradual movement away from the conventional unidirectional model of care (provider to patient) toward a bidirectional model with direct patient engagement and shared decision making over the last decade [31], it is not surprising that many patients perceived completion of a previsit questionnaire as a natural part of the health care interaction. Not only do studies suggest that a majority of patients would prefer to have an active role in their health care [32] but that such participation is associated with improved health outcomes [33]. This perceived responsibility was complemented by the perceived benefits of completing the questionnaire (beliefs about consequences). Some participants believed that completing the questionnaire before their medical appointment would benefit their physician by facilitating history taking and saving time. This finding is similar to that of Howell et al [27], who reported that a desire to reduce consultation time and enhance clinic efficiency motivated patients to complete a preoperative tablet-based questionnaire. Other direct benefits of questionnaire use mentioned by our participants included improving their own understanding of their disease and enabling both more personalized care (eg, a personalized AAP) and a higher quality of care. This perception of higher quality care was also the driving factor behind goal setting and prioritizing questionnaire completion (goals). Given that these factors appear to be strong enablers, patient-facing messaging at the time of an e-questionnaire rollout could reinforce a sense of patient responsibility and these myriad benefits of completing such a questionnaire in an effort to drive usage.

Some participants also noted that their own previous experience with poor health outcomes acted as facilitators by enabling them to recognize the importance of completing the questionnaire (reinforcement). Correspondingly, previous work has shown that perceived health threats predict patient intentions to use smartphone-based health technology [34]. This facilitator may be particularly relevant in diseases that are characterized by episodic flares, such as asthma [35], chronic obstructive pulmonary disease [36], and congestive heart failure [37] and can also be leveraged in patient-facing messaging to drive usage.

Although the vast majority of participants indicated that they would ultimately complete such a questionnaire (intentions), certain barriers and strategies to address them are worth noting. Some participants were not convinced that their questionnaire responses would ultimately prove useful (optimism). This barrier to uptake has been echoed in previous eHealth literature, particularly among older adults concerned about *getting it wrong* [26]. Similarly, some participants expressed concerns about the stress of completing the questionnaire on time and accurately (emotions). These findings suggest that it would be important for any e-questionnaire to include an option to skip questions, and to include response options such as “I don’t know” or “I’m not sure.” Additional messaging could explain that missing answers and data entry errors are acceptable, that patients are simply being asked to “do their best,” and that clinicians will verify and complete data where required. Another concern raised by one participant was the risk of a data security breach (emotions). Indeed, patient concerns about data security are a well-described barrier to the use of social media [38], mHealth apps [39], and eHealth questionnaires in particular [26,27]. These concerns can be addressed upfront through transparent terms of use, privacy, and data policies as well as through opt-out clauses for users. Ideally, a formal privacy impact assessment and threat risk assessment should be performed. Although the cost of formal analyses may be prohibitive for small organizations, some jurisdictions are facilitating this process for developers, such as the National Health Service (NHS) in the United Kingdom, through the NHS Health Apps Library [40].

### Practice Implications

Our findings consisted mostly of enablers to using the e-questionnaire and were identified across participants, and many were supported by the literature in parallel areas. On the basis of these findings, we were able to suggest simple solutions that might drive questionnaire usage. A summary of these identified barriers and enablers and corresponding optimization strategies to address and leverage them are provided in Table 3. These strategies are rooted in concepts related to behavior change techniques (BCTs); however, as a next step, we will employ a formal process (ie, a behavior change matrix) to identify BCTs that will be mapped onto these relevant TDF domains (ie, modifiable barriers and enablers) [41,42].

**Table 3:** Practice implications for uptake and completion of the Electronic Asthma Management System questionnaire based on identified enablers and barriers.

Identified barrier and enabler	Corresponding system optimization strategy
Competing priorities may impede questionnaire completion before appointment (barrier: environmental context and resources)	Reinforce that questionnaire takes only 5 to 10 min to complete and can be done at any time before appointment Provide a reminder to administrative staff at the time of appointment to identify patients who have not completed the questionnaire and prompt them to complete it in waiting room
Preference to complete questionnaire on a larger device (barrier: environmental context and resources)	Develop a web-responsive version of the questionnaire, optimized for completion on large tablets, laptops, and desktop computers
Questionnaire completion will enhance one’s understanding of one’s own disease control (enabler: beliefs about consequences) Questionnaire completion would not help one to better understand their asthma (barrier: beliefs about consequences)	Position the questionnaire app as part of a general educational tool for asthma by developing dedicated web-based educational content that users can access simultaneously through the same app
Prior experiences of poor disease control motivated questionnaire completion (enabler: reinforcement) Questionnaire completion would not lead to better asthma control (barrier: beliefs about consequences)	Include patient-facing messages describing the personal impact of poor disease control, how doing the questionnaire will lead to an AAP^a^, and how an AAP has been shown to reduce symptoms of poor control Enhance the questionnaire app by providing access to the AAP itself through the app
It is part of one’s responsibility to complete the questionnaire to enhance their care (enabler: social/professional role and identity) Questionnaire completion leads to the physician having a more thorough understanding of one’s condition, translating into better health care and disease control (enabler: beliefs about consequences)	Ask each user’s *own* health care provider (physician or nurse practitioner) to send a message to patients emphasizing the following: This is a role that they would like the patient to play in their own care Completing this will enable them to better understand the patient’s disease and to offer better care
Concerns about web-based security of questionnaire data (barrier: emotions)	Conduct a formal privacy impact assessment and threat risk assessment and clearly communicate this robust security approach to users
Concerns about whether one can complete the questionnaire correctly (barrier: emotions)	Provide patients with options to skip questions and responses and ensure availability of telephone and email technical support
A reminder would ensure that the questionnaire would be completed (enabler: behavioral regulation, memory, attention and decision processes; environmental context and resources)	Include *friendly* reminders via automated phone call, email, or text message to complete the questionnaire (and allow personalization of reminder preferences at the time of patient registration)

^a^AAP: asthma action plan.

### Limitations

Our study applied a validated, theory-based methodology to identify and classify barriers and enablers and sampled a diverse population (varied ages and smartphone operating system types). Smartphone ownership is approaching universality in industrialized nations, and we expect that most patients would complete such a questionnaire on a smartphone; however, our results are not pertinent to users without a smartphone who would have to access the system through a web-based, web-responsive questionnaire at home or on a tablet device made available for this purpose in the clinic setting. Recruitment of participants from a quaternary care center may also limit generalizability to other settings. Given that patients with asthma prefer a collaborative role in managing their illness, across a range of asthma severity levels [43], we do not believe that the severity of the disease itself would influence the barriers and enablers that we identified, with the exception of previous poor health outcome experiences acting as a facilitator.

### Conclusions

In this study, we formally identified barriers and enablers in the uptake of a patient-facing mHealth questionnaire. We believe that our findings are broadly relevant to the rapidly growing use of e-questionnaires across health disciplines. Where possible, we suggested strategies that can be used to address barriers and leverage enablers. Future studies are recommended to employ a formal process (ie, a behavior change matrix) to identify behavior change strategies and techniques that are mapped onto the relevant TDF domains (ie, modifiable barriers and enablers) [41,42] and test their impact on actual questionnaire uptake.

## Appendix

Multimedia Appendix 1 Relevant theoretical domains framework domains, belief statements, and sample quotes.

## Abbreviations

AAP: asthma action plan
BCT: behavior change technique
CDSS: computerized decision support system
eAMS: Electronic Asthma Management System
EHR: electronic health record
mHealth: mobile health
NHS: National Health Service
TDF: Theoretical Domains Framework
