# Supplementary material for: Barriers and Enablers to Using a Patient-Facing Electronic Questionnaire: A Qualitative Theoretical Domains Framework Analysis
Source: J Med Internet Res. 2020 Oct 8;22(10):e19474. doi: 10.2196/19474 (PMC7582145; doi:10.2196/19474)
Supplement: Multimedia Appendix 1 [file jmir_v22i10e19474_app1.doc]

Multimedia Appendix 1. Relevant theoretical domains framework domains, belief statements, and sample quotes

| **Domain** | **Belief statements** | **Sample quote (Participant ID)** | **Number of participants out of 12** |
| --- | --- | --- | --- |
| **Knowledge** | I do not need any  further information  about the  questionnaire in order  to complete it | I^a^: “So having seen this questionnaire, do you think you need more information on how to complete it?”  R^b^: “No…I think it was pretty straight-forward.” (1008) | 10 |
|  | It would be helpful to have an advance warning about what information will be required for the questionnaire (e.g. medication list) | R: “I don’t always think about what medication is, perhaps at the beginning you may say something like, “you know we will be asking you about your medications, if you’re not sure what it is, you might want to have it with you…” (1008) | 2 |
| **Skills** | I do not need/do need training to complete the questionnaire | I: “What kinds of training if any would have been helpful to complete this questionnaire? “Do you think you would have needed any training?”  R: “No I don’t think so. It’s pretty straight-forward, you know we’re pretty computer tech savvy now.” (1008)  R: “I think you just go over it with the patient at least once.”(1011) | Do not need (8)/need (1) |
| **Social Professional Role and Identity** | It is/it is not my responsibility to complete the questionnaire | I: “ Do you feel it’s part of your responsibility to fill out this type of questionnaire out before you see the doctor?”  R: “I feel it is because the way I look it, the more information my doctor has about what’s going on with me, the better he or she is able to help me manage my symptoms and cope.” (1004)  R: “No I don’t think it’s my responsibility….if it’s a must than I will, you know, will be forced to do that but I don’t think it’s my responsibility.”(1006) | Is my responsibility (10)/Is not my responsibility (1) |
| **Beliefs about Capabilities** | The questionnaire was easy (9), straightforward (4), concise(1) to complete | R: “It was easy. It was pretty straight-forward. I could understand everything.” (1008) | 11 |
|  | I am confident in my ability to complete the questionnaire | I: “So how confident were you in your ability to complete this questionnaire?” R: “High.”(1002) | 9 |
| **Beliefs about Consequences** | Completing the questionnaire will provide/will not provide my doctor with useful information about my asthma control | R: “…pulling information from people can be very difficult and I think it helps to clarify what our triggers, how much you’re taking, how much they’re not taking and they probably should be taking, all of these questions would be in black and white for the clinician.” (1002)  R: “I don’t see it as something that’s, you know useful, you know for the doctor or myself cause he would already have that on his profile about me.”(1006) | Will provide (9)/will not provide (1) |
|  | Completing the questionnaire helps me/does not help me to understand/reflect upon my asthma control | R: “I think from the patient’s perspective, it would give them more insight as to what’s happening because they actually have to think about what their symptoms are and what they’re doing to help the asthma, so therefore, they can bring that information to the doctor.” (1008)  R: “So for me, personally it wouldn’t make a big difference because I have a good record of my health before I go to see the doctor.”(1009) | Helps me (8)/does not help me (3) |
|  | Completing the questionnaire may lead/may not lead to better asthma management/asthma control | I: How likely do you think it is that completing this questionnaire will lead to better management of your asthma?  R: “Oh, I think definitely. I know if there was something around like this years ago when my asthma was out of control, I think it would have really helped to hone in on where, what’s the problem.” (1002)  I: “Do you think it would be likely that completing the questionnaire like this could lead to better control of your asthma or fewer symptoms for you?”  R: “Not sure I see why that would be case because the choice of going once a year to see Dr. [X], he knows what I’m taking, he asks pertinent questions and then adjusts the medications as he feels is appropriate.”(1009) | May (8)/may not (3) |
|  | Completing the questionnaire will save time for my doctor | R: “It would save a lot of time when you’re in seeing the doctor. All of the questions would be right there in front of them, make it a lot easier for the doctor to assess.” (1002) | Will save (4)/will not save(1) |
|  | Completing the questionnaire will help to inform my asthma action plan | R: “Well I think this one would probably be right at the top because if it’s information that my doctor needs in order to let’s say help put my asthma action plan together, or if he wants to sit down and have a detailed discussion with me as to what should be in my asthma action plan and all that. Once the doctor has all that information in front of him or her, I feel that whatever answers are in that questionnaire could be properly used to set up an asthma action plan.” (1004) | 3 |
|  | Completing the questionnaire will facilitate patient centred/personalized care | R: “The fact that the information is helping the doctor work with me to create a treatment plan that reduces or eliminates my asthma condition or symptoms and overall affect on me. That would definitely influence me to continue to answer these questions.”(1008) | 2 |
| **Reinforcement** | Having had serious health issues, I recognize the importance of completing the questionnaire | R: “Well when I first got diagnosed and before, this is long before this questionnaire came out, I had dealt with two very serious incidents of pneumonia and both times it was 11 days. As I was trying to recover the first time, I said to myself, “if only they would come out with a questionnaire that allows patient to kind of put a number or sign, a letter to how they’re feeling…” (1004) | 4 |
| **Intentions** | I intend/do not intend to complete the questionnaire | I: “Is completing the questionnaire something you plan to do if you're asked to complete it in the future?”  R: “Yes.” (1011)  R: “If I have the choice not to fill it, I will not fill it out, you know. I would maybe fill it out one time, that one time that my doctor should have that on his profile.” (1006) | Intend to (10)/do not intend to (1) |
|  | I would complete the questionnaire if it was close to the time of my appointment | R: “…if my appointment is in a month, maybe I would do it. Maybe if my appointment was in 10 months, I wouldn’t do it.” (1009) | 1 |
| **Goals** | Completion of the questionnaire is a priority/is not a priority for me because the quality of my health care will improve | I: “Is completing the questionnaire something you would plan to do if you were asked to do it in the future?”  R: “Definitely. I would try hard to do it the day before the appointment.” (1010) | Is a priority (11)/is not a priority (1) |
| **Memory, Attention and Decision Processes** | I would need a reminder in order to complete the questionnaire | R: “I honestly think if a person has an appointment booked, and then you send them an email saying your appointment is on such and such date, please fill out this questionnaire to improve the efficiency of your appointment, I can’t imagine people wouldn’t do that.” (1007) | 11 |
|  | I might forget/might not forget to complete the questionnaire due to other issues (e.g. work, family, no symptoms of asthma, reminder sent too far in advance) | I: “Would you remember to fill out the questionnaire?”  R: “You may forget to fill it out. I mean, with society pressure, you know. With everything, with work, with, you know just your family, with just everything and you being sick maybe most of the time, I can speak of myself, you know, I think I would forget that I have to fill it out.” (1006)  R: “I’d probably do it like 2 weeks before, considering I have access to my smartphone.” (1004) | Might forget (5)/might not forget (1) |
| **Environmental Context and Resources** | A friendly reminder from the doctor’s office would ensure that I complete the questionnaire  *Desired reminder format:  email (8); SMS text message (5); phone call (1) | R: “If it’s a friendly reminder from you guys to fill out the questionnaire, of course someone like me, I’d be receptive and I’ll be like okay I’m being reminded to fill out, better go do it.” (1004) | 11 |
|  | There were technical/resource issues/were no technical/resource issues that might influence whether I complete the questionnaire | R: “Well I found that if you just fill out the information in the field, rather than use the drop downs that it worked much easier.”  I: “So that drop downs were a bit more….”  R: “Cumbersome….” (1002)  I: “Do you think there’s anything that would influence you, in terms of whether or not you’d be able to complete the questionnaire, in terms of the equipment you might need or technical issues, Wi-Fi, competing tasks?”  R: “I can’t think of anything.” (1010) | Were technical/  resource issues (3)/were no technical/  resource issues (6) |
|  | Lack of time/other priorities might prevent me from filling out the questionnaire | R: “I have my two personal emails. I have 4 different work emails that I have to go through every day. Like it’s swamped with these things.” (1003) | 4 |
|  | The ability to complete the questionnaire on my own time, in a relaxed, comfortable space would make me more likely to complete it | R: “I think it’s more relaxed which makes the patient feel easier and there’s no rush. Like okay, you’re doing the questionnaire and somebody comes to the door or you can just go answer the door, and then go back to the questionnaire, or be doing the questionnaire at 3 o’clock in the morning if you want…” (1003) | 3 |
|  | I believe the questionnaire would have been more accessible via a larger device (e.g. computer/iPad instead of a phone) | R: “I’m pretty competent on the internet and maybe on an iPad like on a larger device it might have been more free flowing.”(1002) | 4 |
|  | Having pictures/lists of respiratory medications helped me to complete the questionnaire | R: “the pictures for the medicine, that was really good because I’m sure that a lot of people don’t really like know all the details of their medications so that was really nice.”(1009) | 3 |
|  | Having telephone or in person assistance would make me more likely to complete the questionnaire | R: “I know that there’s no time in the doctor’s office to put you through it but I do think it would help people that aren’t computer savvy to have someone run through it with them once.” (1002) | 2 |
|  | I would be more likely to complete such a questionnaire if it were on paper | R: “I personally prefer paper.”(1005) | 2 |
| **Social Influences** | Health professionals (e.g. the doctor or someone from the doctor’s office) would influence me to complete the questionnaire | I: “Who would influence you to complete the questionnaire?”  R: Who? Well I suppose the doctor’s office if they sent out a text to me or something. I can’t, I don’t think anyone else would influence me.” (1002) | 6 |
|  | No one would influence me to complete the questionnaire | I: “… in your circle or personal circle, who might influence whether or not you would complete this questionnaire at all?”  R: “Nobody. It would be my choice.” (1008) | 5 |
|  | My family members would influence me to complete the questionnaire | R: “Gosh if my family members knew of it they would certainly encourage me to or push me to fill it in.” (1010) | 3 |
|  | Perceived needs of the healthcare system would influence me to complete the questionnaire | R: “If the system sees the need for me to do this in order to get the healthcare that I need, and therefore, I would do it.” (1007) | 1 |
| **Emotions** | I have/do not have concerns/worries about completing the questionnaire | I: “Are you worried or have any concerns about completing the Questionnaire at all?”  R: “No.”(1007)  R: “My biggest worry is whether I can get it done before I get to see the doctor.” (1008)  R: “Medical information, security is a big deal.” (1009)  R: “I guess there's like always a certain level of like oh, did I do it right or did I do it, like did it go through?”(1012) | Have concerns/  worries (5)/do not have concerns/  worries (6) |
| **Optimism** | I am not confident that my questionnaire responses will be useful | R: “I think for average people, I’m not sure that the results would be reliable to be so very useful. I imagine that regardless of what’s on the survey, that the doctor would verify all of it with me again anyway…” (1009) | 2 |
| **Behavioural Regulation** | Using my calendar, a reminder on my phone and/or email will ensure that I complete the questionnaire | R: “Yeah so I’d get the email, if I try to do it right away if I can; if not I would definitely put maybe a reminder like if the questionnaire says have it done before a week before I’d put it in like two or three days before have it due so that I have it entered by the time I have to have it done.”  I: “So would you kind of put a reminder in your phone to let you know…?”  R: “Yes. I’d put a reminder in my phone.” (1008) | 7 |

^a^I: Interviewer

^b^R: Respondent
